# In this issue: Volume 114, Issue 12, December 2023

**DOI:** 10.1111/cas.16022

**Published:** 2023-11-22

## Intratumoral administration of unconjugated Accum™ impairs the growth of pre‐established solid lymphoma tumors

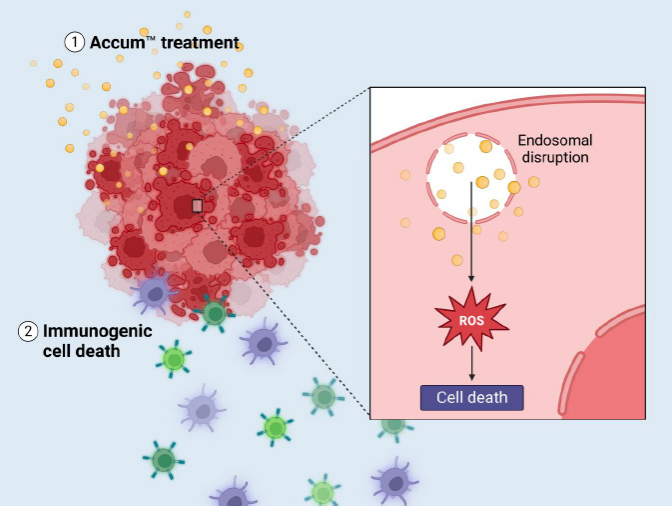

With the evolution of cancer research, targeted anticancer therapies like antibody–drug conjugates, immune checkpoint inhibitors (ICIs), cellular cancer vaccines, and adoptive cell therapies have been introduced. These targeted therapies, however, face one roadblock: intracellular pathways interfere with their deposition inside a cell and reduce drug activity. To overcome this barrier, the Accum™ technology was developed.

Accum™ (which consists of a cholic acid linked to a nuclear localization peptide sequence) helps the conjugated therapeutic molecule to escape entrapment in endosomes (organelles that regulate the transport of molecules within a cell) and thus, accumulate in designated cells. In this study, Bikorimana et al. sought to understand if the unconjugated Accum™ molecule could innately kill cancer cells. They used mouse cell lines, molecular assays, and drug therapy assays to understand the anticancer activity of Accum™.

Apart from acting cohesively with common ICIs, unconjugated Accum™ could halt tumor progression when delivered to a tumor. The researchers also found that unconjugated Accum™ exhibited cytotoxic activity and induced cell death in mouse tumor cell lines. This was attributed to the production of reactive oxygen species, induction of immunogenic cell death, and the innate capacity of Accum™ to disrupt endosome membranes at the molecular level.

Interestingly, when Accum™ was administered as a stand‐alone therapy to animals with cancer, the molecule recruited CD4^+^ and CD8^+^ T cells (that play an important role in immune response) to the tumor. Moreover, Accum™ was compatible with three ICIs (anti‐CTLA4, anti‐PD‐1, or anti‐CD47), but its activity was not enhanced when it was combined with all three.

This is the first study to show that unconjugated Accum™ harbors an innate anticancer activity. It thus paves the way for developing new anticancer drugs based on the cancer killing properties of unconjugated Accum™ variants.

https://onlinelibrary.wiley.com/doi/full/10.1111/CAS.15985

## Gut microbiome as a biomarker for predicting early recurrence of HBV‐related hepatocellular carcinoma

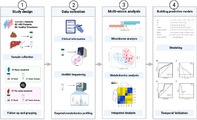

Microbes in the gut play a crucial role in controlling the host body's metabolism, inflammation, and immune response. As a result, the ecosystem of microbes in the gut, known as the ‘microbiome,’ has a major impact on various liver diseases, including liver cancer. Hepatocellular carcinoma (HCC) is the most common liver tumor, accounting for 85%–90% of all liver cancer cases. Infections of hepatitis B virus (HBV) and hepatitis C virus (HCV) are responsible for the majority of HCC cases globally. The preferred treatment for HCC is complete tumor removal, but the cancer often recurs after surgery, making full recovery difficult.

This study found an association between the composition of gut microbes and the early recurrence of HCC after surgery. The study included 124 patients diagnosed with HBV‐associated HCC, 82 with HBV‐related hepatitis, and 86 healthy volunteers. The study results indicate that bacteria from *Dialister, Veillonella*, the *Eubacterium coprostanoligenes* group, and *Lactobacillus* genera, as well as the *Streptococcus pneumoniae* and *Bifidobacterium faecale* species, are linked to early HCC recurrence. Furthermore, specific chemical compounds produced by gut microbes, including acetic acid, glutamate, and arachidonic acid, were also associated with early recurrence. By extensively analyzing the diversity of the gut microbiome and the chemicals present in the gut and liver, this study further suggests that acetic acid produced by gut microbes might provide energy for liver tumors to grow again, leading to recurrence.

The research suggests that the composition of gut microbes can serve as valuable indicators or “biomarkers” to predict the likelihood of early HCC recurrence. This information could be extremely beneficial for healthcare professionals as it allows them to identify patients at a higher risk of HCC recurrence. By shedding light on a potential mechanism through which gut microbes contribute to the early recurrence of HCC, this study could open doors for the development of new treatments or interventions aimed at preventing or managing the recurrence of liver cancer.

https://onlinelibrary.wiley.com/doi/full/10.1111/CAS.15983

## DYRK2 promotes chemosensitivity via p53‐mediated apoptosis after DNA damage in colorectal cancer

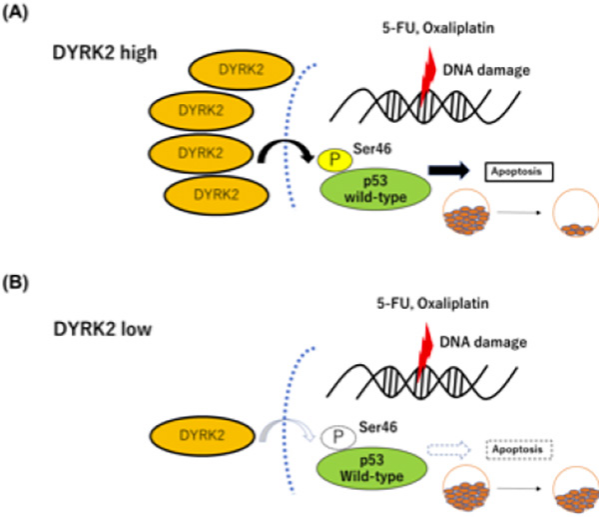

Colorectal cancer (CRC) is one of the leading causes of cancer‐related deaths worldwide. The outcomes for patients with advanced CRC are often poor because of the cancer's ability to metastasize (spread to other locations in the body) and/or recur. Chemotherapy, while effective in reducing recurrence and eliminating residual disease, presents a conundrum: patients in the same stage of CRC who receive the same treatment often experience different prognoses. Identifying robust predictive markers and therapeutic approaches for chemotherapy would go a long way in improving the survival rates of patients with CRC.

Dual‐specificity tyrosine‐regulated kinase 2 (DYRK2) is a protein kinase that can trigger cell death in response to DNA damage, primarily by acting on p53 within the cell nucleus. Although DYRK2 has tumor‐suppressing capabilities, its relationship with chemotherapy‐induced DNA damage in CRC remains unknown. In this study, Takano et al. explored DYRK2's role as a potential marker to gain deeper insights into how two chemotherapeutic agents, 5‐fluorouracil and oxaliplatin, work.

The researchers found that when DYRK2 was removed from human CRC cells by gene knockout, the cells became less responsive to 5‐fluoroucil and oxaliplatin treatment, but only if the CRC cells had wild‐type p53. However, when researchers artificially increased DYRK2 expression in CRC cells with wild‐type p53, cell death increased, and the cells became more sensitive to chemotherapy. The effect was mainly due to p53‐Ser46 phosphorylation. To further understand the impact of DYRK2 knockout, researchers designed a xenograft model in which parental CRC cells or those lacking DYRK2 were implanted into mice. They found that the tumors derived from CRC cells lacking DYRK2 were less sensitive to treatment with 5‐fluorouracil and oxaliplatin than those derived from parental CRC cells.

These results show that DYRK2 expression is intricately linked to the sensitivity of CRC cells treated with 5‐fluorouracil and oxaliplatin, and p53 has a role in mediating this response. Therefore, by evaluating the levels of DYRK2 and p53 in CRC patients, healthcare providers can better determine treatment strategies for CRC chemotherapy. This research takes a step closer to personalizing treatment plans for CRC patients, potentially leading to more effective and targeted therapy, and fewer mortalities.

https://onlinelibrary.wiley.com/doi/full/10.1111/CAS.15973

